# Effect of Linseed Feeding on Carcass and Meat Quality and Intramuscular Fatty Acid Profile of Simmental Bulls Slaughtered at Different Ages

**DOI:** 10.3390/foods14071098

**Published:** 2025-03-21

**Authors:** Ana Kaić, Dubravko Škorput, Zoran Luković, Krešimir Salajpal, Kristina Kljak, Nives Marušić Radovčić, Danijel Karolyi

**Affiliations:** 1 Division of Animal Science, Faculty of Agriculture, University of Zagreb, Svetošimunska Cesta 25, 10000 Zagreb, Croatia; akaic@agr.hr (A.K.); dskorput@agr.hr (D.Š.); lukovic@agr.hr (Z.L.); ksalajpal@agr.hr (K.S.); kkljak@agr.hr (K.K.); 2 Faculty of Food Technology and Biotechnology, University of Zagreb, Pierottijeva 6, 10000 Zagreb, Croatia; nmarusic@pbf.hr

**Keywords:** beef, slaughter weight, meat quality, n-3 polyunsaturated fatty acids, lipid oxidation

## Abstract

The inclusion of linseed in cattle diets can improve the fatty acid (FA) profile of meat, but the effects of supplementation at different ages have been insufficiently studied. Hence, this study examined the effects of linseed supplementation and slaughter age on beef cattle carcass and meat quality, FA profile, and lipid oxidation. Eighty Simmental bulls (initial age 221 ± 9 days) were evenly allocated the experimental treatments, consisting of a control diet and a linseed-enriched diet (around 1% whole linseed) fed to the bulls until slaughter at 13 or 17 months of age, in a 2 × 2 factorial design. After slaughter, carcass traits, meat quality, FA profile, and oxidative stability (TBARS test) of the longissimus thoracis muscle were determined. Linseed feeding increased the dressing percentage (*p* < 0.01) at both slaughter ages but had limited effects on overall carcass and meat quality. The intramuscular fat of linseed-fed bulls contained less saturated FA (*p* < 0.01) and more beneficial n-3 PUFAs, such as eicosapentaenoic (*p* < 0.05) and α-linolenic acid (*p* < 0.001), especially in younger bulls. Although the atherogenic index and n-6/n-3 ratio improved, they remained above the recommended values. Lipid oxidation was higher in linseed-fed (*p* < 0.05) and younger bulls (*p* < 0.001). These results suggest that linseed supplementation can improve the beef’s FA composition, but higher levels, especially in older animals, and antioxidant strategies may be required to optimise meat stability and nutritional value.

## 1. Introduction

Improving carcass and meat quality traits in beef production is crucial to meet the changing demands of consumers who are looking not only for high-quality meat but also for products that are in line with health-conscious and sustainable dietary choices [1,2,3]. Carcass traits, such as muscle conformation, fat cover, and marbling, are key factors that influence the economic value of beef cattle as they directly affect meat yield and quality grading [1,4]. In addition, colour, intramuscular fat content, fatty acid profile, and oxidative stability significantly shape consumer purchasing decisions and influence the shelf life and attractiveness of meat and meat products [1,2]. Advances in nutritional strategies, such as linseed supplementation, offer promising approaches to improve these parameters while increasing the nutritional value of beef.

Beef is an important source of saturated fatty acids (SFAs), comprising about 40% SFAs, 50% monounsaturated fatty acids (MUFAs), 5% trans fatty acids, and 4% polyunsaturated fatty acids (PUFAs) [5]. Numerous studies have linked high dietary intake of SFAs to increased health risks, including cardiovascular disease and cancer [6,7]. In contrast, emerging evidence suggests that a diet rich in PUFAs, particularly omega-3 fatty acids and conjugated linoleic acids (CLAs), may provide health benefits by lowering low-density lipoprotein (LDL) and cholesterol levels and supplying anti-inflammatory, anti-atherogenic, and anti-carcinogenic effects [8]. Linseed supplementation has been shown to be effective in modifying the fatty acid profile of beef to make it more health-promoting by increasing the content of beneficial omega-3 PUFAs [9,10,11]. As a source of alpha-linolenic acid (ALA), linseed enriches beef with ALA, which serves as a precursor to longer-chain omega-3 fatty acids such as eicosapentaenoic acid (EPA) and docosahexaenoic acid (DHA) [7]. Studies have shown that despite the significant biohydrogenation of dietary PUFA carried out by rumen microbiota [12], the consumption of linseed can significantly increase the omega-3 content of muscle tissue while improving the overall PUFA-to-SFA ratio, which is beneficial for health [2,3]. This approach not only meets consumer demand for healthier meat products but also addresses the less favourable fatty acid composition often found in conventionally produced beef [13].

Recent studies emphasize the importance of nutritional interventions that influence not only the fatty acid composition of meat but also broader aspects of carcass and meat quality. For example, linseed supplementation has been found to enhance various organoleptic properties of beef, such as decreasing fat odour and enhancing beef flavour [14]. It also promotes fat deposition [15] and increases the antioxidant capacity of muscle by supplying natural antioxidants, thereby reducing lipid oxidation during storage [16,17]. In addition, optimizing the balance between SFAs and unsaturated fatty acids (UFAs) through strategic nutritional interventions, such as linseed enrichment, supports the production of meat with improved nutritional profiles, which meets the growing consumer demand for healthier animal products [2,3,18].

Oxidative stability is another critical aspect of meat quality that is becoming increasingly important. Lipid oxidation is a major factor in the deterioration of the colour, flavour, and nutritional value of meat and leads to a shorter shelf life [19,20,21]. As PUFAs are more susceptible to oxidation, the addition of antioxidants or application of a diet rich in natural sources of these fatty acids and antioxidants, such as linseed, is a strategic approach to maintaining meat quality. Linseed is not only rich in ALA, an omega-3 fatty acid, but also contains natural antioxidants such as lignans, which can improve the oxidative stability of meat products [22,23]. Studies have shown that feeding with linseed can reduce the rate of lipid oxidation in meat, thereby maintaining meat quality during storage [16,24]. Its ability to improve nutritional quality while extending shelf life makes linseed supplementation an effective strategy for producing high-quality, healthy fatty-acid-enriched beef.

As beef production is increasingly moving towards more sustainable and health-oriented practices [25], dietary interventions such as linseed supplementation represent a practical and versatile approach to optimising carcass and meat quality [17,26]. While the beneficial impact of linseed supplementation on beef quality are well documented, there is a lack of research investigating how age-related factors affect the outcomes of dietary supplementation across different age groups. To address this gap, this study aimed to investigate the effects of linseed-enriched diets on the carcass traits and meat quality of young Simmental bulls at two distinct stages of growth: younger animals used as yearlings for the production of so-called “baby-beef” and older animals typically finished for conventional beef production, with a focus on fatty acid composition and lipid oxidation stability.

## 2. Materials and Methods

The study was conducted in full compliance with Croatian legislation, including the Animal Protection Act (Official Gazette 102/17) and the Regulation on the Protection of Animals Used for Scientific Purposes (Official Gazette 55/13). Ethical approval was obtained from the Bioethical Committee for the Protection and Welfare of Animals at the University of Zagreb, Faculty of Agriculture, Croatia (Class: 114-03/25-02/01; Ref. 251-71-2-02/19-25-2, issued on 9 January 2025).

### 2.1. Animals, Study Design, and Diets

The study involved 80 Simmental bulls reared on a large commercial beef farm as part of an intensive production system. The animals were divided equally (n = 20) into experimental treatments in a 2 × 2 factorial design, consisting of a control diet and a linseed-enriched diet (around 1% whole linseed), fed to the bulls until slaughter at the age of 13 months (n = 40) or 17 months (n = 40). The average (± SD) initial age and live weight per diet of each slaughter-age group were: 222.3 ± 7.0 days and 310.1 ± 15.8 kg in the linseed-fed group slaughtered at 13 months of age (L13), 222.0 ± 9.0 days and 311.6 ± 14.4 kg in the control-fed group slaughtered at 13 months of age (C13), 221.1 ± 8.5 days and 274.5 ± 12.7 kg in the linseed-fed group slaughtered at 17 months of age (L17), and 218.2 ± 9.3 days and 282.6 ± 10.1 kg in the control-fed group slaughtered at 17 months of age (C17). All groups were housed freely in separate pens with a concrete floor covered with straw, providing 2.8–4.3 m^2^ per animal. The diets were offered as a total mixed ration (TMR) containing high-moisture maize, maize silage, hay, and a high-protein supplement of soya meal and rapeseed meal, as well as a mineral and vitamin mixture (Table 1). In the diet enriched with linseed, part of the high-moisture maize was replaced with whole-grain linseed. The TMR was fed to the bulls once daily in the morning. At the start of the feeding trial, all bulls received 4.4 kg of high-moisture maize (or 4.2 kg + 0.13 kg linseed for the L diet), 6.0 kg of maize silage, 1.5 kg of the protein supplement, and 0.2 kg hay per animal per day. Up to 13 months of age (164 feeding days), the amount of high-moisture maize in all groups was gradually increased to 7.0 kg (or 6.8 kg + 0.13 kg linseed for the linseed-enriched diet), while maize silage was increased to 8.5 kg per day. The bulls in groups L13 and C13 were slaughtered at the age of 13 months. For the L17 and C17 bulls at 13 to 17 months of age (129 feeding days), the high-moisture maize was increased to 8.0 kg (or 7.8 kg plus 0.16 kg linseed for the linseed-enriched diet), and the maize silage was increased to 9.0 kg per day. The amounts of protein supplement and hay remained constant throughout the trial. The average ingredient contents throughout the feeding periods and chemical composition of the diets given to the bulls during the trial are shown in Table 1. The average chemical and fatty acid compositions of the feeds used are given in Supplementary Materials Table S1.

### 2.2. Measurements and Sample Collection

The bulls were slaughtered either at 13 months of age (L13 and C13 groups) or at 17 months of age (L17 and C17 groups) according to standard procedures in an EU-approved slaughterhouse located about 10 km from the farm. Immediately after slaughter, the weight of each hot carcass was recorded, and the dressing percentage was calculated. On the same day, the carcasses were categorized for conformation and fat content (EUROP system) according to the EU Regulation No. 2017/1182 [27]. The pH value of the *M. longissimus thoracis* (LT) was measured on the right half-carcasses between the 12th and 13th rib 45 min after slaughter (pH_1_) using a portable pH meter (TESTO 230, Testo SE & Co., KGaA, Titisee-Neustadt, Germany) with a penetration glass probe. The pH meter was previously calibrated according to the manufacturer’s instructions with Testo buffer solutions with pH values of 4.00 and 7.00. After chilling the carcasses for 24 h at +4 °C, the pH value (pH_2_) was measured again on the same side of the LT muscle using the same method. Muscle colour was determined 24 h postmortem on the same LT muscles using a Minolta CR-410 chroma meter (Konica Minolta Sensing Inc., Osaka, Japan), which was calibrated in D_65_ illuminant with a standard white ceramic tile (Y = 94.5, x = 0.3158, y = 0.3323) before measurements were taken. Colour was expressed using the CIE L*a*b* system, which measures lightness (L*), redness (a*), and yellowness (b*). The measurements were taken on the cut surface (cranial direction) of the LT muscle between the 12th and 13th rib after approximately 15 min of blooming time. Three measurements were taken for each LT, and the mean value was used for further analysis. After colour assessment, samples of LT muscle were taken at the level of the last rib and frozen at −20 °C for subsequent analysis of chemical composition, fatty acid (FA) composition (total lipids), and lipid oxidation.

### 2.3. Chemical Analysis

The moisture, ash, protein, and fat content of LT samples (13th rib level) were analyzed according to the standard methods for meat and meat products [28,29,30,31]. The composition of FAs of total lipids was determined by gas–liquid chromatography using the in situ transesterification method [32]. The content of fatty acid methyl esters (FAMEs) was analyzed using an Agilent Technologies 6890 N gas chromatograph (Agilent Technologies, Santa Clara, CA, USA) equipped with a flame ionization detector and a Supelco Omegawax™ (MZ-Analysentechnik GmbH, Mainz, Germany) 320 capillary column (30 m length, 0.32 mm inner diameter, 0.25 μm film thickness) for FAME separation. The FAMEs were identified by comparing their retention times with those of a standard mixture (Nu-Check Prep, Inc., Elysian, MN, USA), which was also used to determine the response factor (Rf) for each FA. The mass fraction of each FA was calculated using the Rf and a conversion factor from FAME to FA content.

Lipid oxidation was assessed on thawed LT muscle samples that were homogenized, wrapped in oxygen-permeable polyethylene film, and stored at +4 °C. Analyses were performed after 0, 3, and 6 days of cold storage. Lipid oxidation was measured using the 2-thiobarbituric acid-reactive substances (TBARS) assay following a slightly modified version of the method described by Botsoglou et al. [33]. In brief, 2 g of the sample was placed in a 50 mL polypropylene test tube, followed by the addition of 8 mL of 5% aqueous trichloroacetic acid (TCA) and 5 mL of 0.8% butylated hydroxytoluene (BHT) in hexane. The mixture was homogenized at high speed for 30 s (Ika T10 basic, Ultra Turrax, IKA-Werke GmbH & Co. KG, Staufen, Germany), and then centrifuged at 2500 g for 5 min (Centric 322A, Tehtnica, Železniki, Slovenia). The upper hexane layer was discarded, and the bottom aqueous layer was adjusted to a volume of 10 mL with 5% TCA. If necessary, the reaction mixtures were filtered (grade 391; Munktell & Filtrak GmbH, Bärenstein, Germany). A 2.5 mL aliquot of the TCA solution was transferred to a polypropylene tube with a screw cap, and 1.5 mL of 0.8% aqueous 2-thiobarbituric acid (TBA) was added. The mixture was incubated at 95 °C (WB22, Memmert GmbH + Co. KG, Schwabach, Germany) for 30 min and then cooled under tap water. The absorbance was measured at 532 nm (Helios γ, Thermo Electron Corporation, Waltham, MA, USA) against a blank containing 2.5 mL of TCA and 1.5 mL of 0.8% TBA. The malonaldehyde (MDA) content was calculated using a standard curve generated with 1,1,3,3-tetramethoxypropane. TBARS values were measured in triplicate and expressed as mg of MDA per kg of tissue.

### 2.4. Statistical Analysis

The data were analysed via an analysis of variance (ANOVA) following a 2 × 2 factorial design using the general linear model (GLM) procedure of SAS 9.4, with the model including age at slaughter, diet, and their interaction as fixed effects, using the following formula:

y_ijk_ =μ + α_i_ + β_j_ + (αβ) _ij_ + ϵ_ijk_

where y_ijk_ is the value of the analysed parameter, µ is the overall mean, α is the effect of slaughter age (i = 1–2), β is the effect of diet (j *=* 1–2), αβ is the interaction of slaughter age × diet, and ϵ_ijk_ is the random error.

If the interaction between age at slaughter and diet was significant, a post hoc comparison of least squares means was performed using the Tukey test. Differences were considered statistically significant if *p* < 0.05.

## 3. Results and Discussion

### 3.1. Slaughter Performance Traits

The effects of age, feed type, and their interaction on the slaughter performance and meat quality traits of Simmental bulls are shown in Table 2.

The results show that slaughter age had a significant effect (*p* < 0.001) on several slaughter parameters in Simmental bulls. As expected, older bulls slaughtered at 17 months of age had better slaughter characteristics compared to younger bulls slaughtered at 13 months of age, including higher final weight (on average by 14%), hot carcass weight (by 16%), dressing percentage (by 1.5 percentage points or 3%), and trimmed fat content (by 41%). These differences can be attributed to the physiological maturity of the older animals, which generally leads to a greater deposition of fat and muscle, which in turn contributes to a higher carcass weight and improved dressing percentages. In addition, slaughter age had a significant effect on EUROP conformation and fatness scores, reflecting the effect of animal maturity on muscle development and carcass quality [34]. Bulls slaughtered at 17 months had higher conformation and fatness scores (by 15% and 5%, respectively) than those slaughtered at 13 months. This difference can be attributed to the prolonged growth period, which allows older bulls to achieve greater muscle development and better carcass shape, which is directly assessed in the conformation scoring systems. However, growth intensity decreases with increasing age, as shown by the higher average daily weight gain of bulls slaughtered at 13 months of age (Table 2). The results in Table 2 show that diet had a significant effect only on the dressing percentage in Simmental bulls (*p* < 0.01). Bulls fed the linseed-enriched diet achieved higher dressing percentage (1.5 percentage points or 2.6% at 13 months of age and 0.7 percentage points or 1.2% at 17 months of the age) compared to those fed the control diet, suggesting improved efficiency in converting live weight to carcass weight. However, linseed supplementation did not significantly (*p* > 0.05) affect other slaughter performance traits, including live weight, slaughter weight, and hot carcass weight (Table 2). These results are consistent with studies by Marino et al. [35] on young Podolian × Limousine bulls, Conte et al. [36] on young Maremmana bulls, Morittu et al. [24] on young Charolaise × Podolica bulls, and Corazzin et al. [9] on young Italian Simmental and Holestein bulls, which also reported no significant effect of linseed supplementation on these traits. However, these studies found no influence of linseed on dressing percentages. In contrast, Albertì et al. [15] observed a significant reduction in dressing percentage when whole linseed was included at a 5% supplementation level in the diet of young Pirenaica bulls, reporting 60.4% for the linseed group vs. 62.6% for the control group. The authors attributed this unexpected decrease to the high mucilage content of linseed, which can absorb large amounts of water, leading to increased gut fill and subsequently reducing carcass yield.

Regarding the EUROP conformation score, there was a significant (*p* < 0.05) interaction between age at slaughter and diet, with a significantly higher score only in linseed-fed bulls slaughtered at 13 months of age (4.35 vs. 3.85, *p* < 0.05), but with no such differences in conformation score between diets in older age group. The fact that the interaction between slaughter age and diet was only significant for conformation scores underlines the need to evaluate these factors together, as their combined effects can optimize carcass traits that directly affect quality and market value, such as muscle conformation.

### 3.2. pH Value and Colour Parameters

The results in Table 2 show that the age at slaughter had no significant effect (*p* > 0.05) on the pH values of the LT muscle. This suggests that the rate of postmortem glycolysis and the ultimate pH were comparable across all age groups, possibly due to the similar pre-slaughter conditions and similar muscle glycogen levels in younger (13 months) and older (17 months) animals. This corroborates the previous results of Marenčić et al. [37], who found no differences in the pH of meat from Simmental bulls slaughtered at 13–14, 15–14, or 17–18 months of age.

Values of pH_2_ between 5.68 and 5.77 are not expected to have a negative effect on meat quality, as only final pH values above 5.8 are generally associated with reduced quality [38]. Diet had a significant effect (*p* < 0.05) on LT muscle pH_1_, with bulls fed the linseed-enriched diet having a lower pH_1_ than the control group (i.e., 6.32 vs. 6.49 at 13 months of age and 6.43 vs. 6.62 at 17 months of the age). This suggests that linseed supplementation may have accelerated postmortem glycolysis, resulting in a faster initial pH drop immediately after slaughter. However, this difference was no longer observed after 24 h, as the final pH values did not differ between the two groups (*p* > 0.05), which is consistent with the results of Corazzin et al. [9].

Colour is one of the most important quality attributes influencing consumer perception of meat, with red hues being strongly preferred over purple or brown tones [39]. Among the colour parameters, redness (a*) is considered the most reliable indicator of meat colour acceptability [40,41]. As summarized by Sánchez et al. [42], beef of acceptable quality must have an a* value of more than 14.5 or 16.8, which is consistent with the results of our study. The lack of significant differences in redness (Table 2) suggests that myoglobin levels, which primarily influence this parameter, were comparable across age groups. However, the results of the present study show that age at slaughter has a significant effect on lightness (L*; *p* < 0.001) and yellowness (b*; *p* < 0.05). Older bulls (17 months) had lower lightness and yellowness values than younger bulls (13 months), suggesting that their meat was darker and less yellow. This is consistent with the results of previous studies in which older age resulted in darker meat, probably due to higher myoglobin content and changes in fat deposition, which can reduce the lightness and yellowness of meat colour [38,43].

Marenčić et al. [37] found that extending the slaughter age of Simmental bulls by four months (from 13–14 months to 17–18 months) resulted in beef muscles with reduced L* values (42.55 vs. 41.78) and slightly increased, although not in a statistically significant manner, a* (29.17 vs. 29.48) and b* (11.38 vs. 11.60) values. Similarly, Sergatini et al. [44] found that increasing the slaughter age of young Maremmana bulls from 18 to 24 months significantly decreased L* values (38.40 vs. 41.10) and increased b* values (6.65 vs. 10.78), while a* values remained unchanged. In contrast, Kopuzlu et al. [45] found that older animals (19, 25, and 27 months) had higher L* values (40.17, 41.42, and 41.92) and a* values (22.85, 22.88, and 22.45) compared to younger animals (15 and 17 months), which had lower L* values (38.13 and 38.47) and a* values (18.99 and 19.46) in East Anatolian Red bulls.

In addition, diet had no significant effect (*p* > 0.05) on LT muscle colour parameters, which remained similar across the control group and the linseed-enriched diet groups. These results are consistent with those of Mach et al. [46], Corazzin et al. [9], and Tarricone et al. [47], who also found that linseed supplementation had no effect on beef colour. On the contrary, Morittu et al. [24] reported a decrease in a* and b* meat colour in Charolaise × Podolica bulls fed a linseed diet (440 g/head/day). There was no significant interaction between age and diet for the pH or colour parameters of LT muscle (*p* > 0.05).

### 3.3. Chemical Analyses

The chemical composition of LT muscle was influenced by both age at slaughter and diet (Table 2). Older bulls (17 months) had a significantly higher intramuscular fat content (on average by 29%) than younger bulls (13 months) (*p* < 0.01), probably due to the natural increase in fat deposition with age and slaughter weight [48]. An increase in the source of starch from corn feeds, which were gradually increased during the feeding trial to meet the cattle’s growing nutritional requirements, may have also contributed to higher intramuscular fat deposition [49]. Intramuscular fat is known to improve sensory characteristics such as tenderness, juiciness, and flavor [43]. In contrast, the results show that moisture and protein content were not affected (*p* > 0.05) by age at slaughter. In line with these results, Bureš and Barton [43] observed an age-related increase in intramuscular fat and dry matter content in Charolais × Simmental bulls and heifers slaughtered at 14 or 18 months of age. However, other studies have reported different observations. For example, Kopuzlu et al. [45] found that slaughter age (between 15 and 27 months) had no significant effect on the dry matter, fat, ash, or protein content of Eastern Anatolian Red bulls. Similarly, Sergatini et al. [44] reported no significant differences in the proportions of moisture, protein, fat, or ash in young Maremmana bulls slaughtered between 18 and 24 months of age.

In the present study, ash content was slightly higher (about 0.5%) in younger bulls (*p* < 0.05). This could be due to a dilution effect, as the greater muscle and fat mass in older animals reduces the relative ash content.

In this study, a linseed-enriched diet administrated at a dose of 130 g or 160 g whole linseed per head per day until 13 or 17 months of age (0.8% or 0.9% of the diet administrated) throughout the fattening period significantly increased protein (*p* < 0.05) and ash content (*p* < 0.05), on average by 1% compared to the control diet, indicating slightly improved muscle protein deposition and higher mineral content. However, Morittu et al. [24] reported that dietary supplementation at a higher linseed dose (440 g/head/day) had no significant effect on the chemical composition of LT muscle in young Charolais × Podolica bulls. In particular, they observed an almost significant variation in intramuscular fat content between groups, which they attributed to the low body weight uniformity of the young, crossbred bulls. In line with these results, Tarricone et al. [47] observed that the chemical composition of the *longissimus lumborum* muscle in young Podolica bulls remained unchanged regardless of dietary treatment (control or 3% linseed supplementation). Corazzin et al. [9] investigated the effects of whole-linseed supplementation (5% and 8% DM of the daily ration) on the LT muscle of young Italian Simmental and Italian Holstein bulls. They reported that linseed supplementation only affected the dry matter content of the muscle, while other components of the chemical composition, such as crude protein, intramuscular fat, and ash, remained unaffected.

The results of the present study show that moisture content remained constant in all groups, indicating stable water retention regardless of age or dietary treatment. The interaction between slaughter age and diet had no statistically significant effect on the chemical composition of LT muscle (*p* > 0.05).

### 3.4. The Intramuscular Fatty Acid Composition

The effects of age at slaughter, feed type, and their interaction on the intramuscular FA composition of Simmental bulls are shown in Table 3.

Regarding the overall FA composition of LT muscle, the most abundant FAs were oleic acid (C18:1) and palmitic acid (C16:0), which together accounted on average for more than 60% of the total FA content, followed by stearic acid (C18:0) with a pooled share of about 17% and linoleic acid (C18:2n-6, LA) with a share of about 8%. The average proportion of myristic acid (C14:0), arachidonic acid (C20:4n-6), and palmitoleic acid (C16:1) was between 2 and 3%, while the proportion of the other FAs was less than 1%. Overall, the shares of total saturated FA (SFA) and monounsaturated FA (MUFA), were around 45% and 43%, while the share of total polyunsaturated FA (PUFA) was about 12%, with average totals of n-6 and n-3 PUFA of 11 and 1%, respectively. Compared to the FA profile of grain-fed beef reported in the literature (e.g., in the review by Muchenje et al. [51]), the observed FA values chiefly correspond to the indicated ranges.

Age at slaughter had a minor effect on FA profile and affected only the concentration of several individual FA, i.e., C17:1 (*p* > 0.05) and eicosapentaenoic acid (EPA) C20:5n-3 (*p* < 0.01) and C22:4n-6 (*p* < 0.01), all of which were present in lower concentrations in bulls slaughtered at 17 months of age (Table 3). In addition, the concentration of CLA, which is determined by gas chromatography as a mixture of c9-t11 and t10-c11 isomers of C18:2, was also influenced by slaughter age (*p* < 0.001) and was higher in older animals, roughly by about 50%.

CLA, a product of the rumen biohydrogenation of unsaturated FA with potential benefits for human health, is a mixture of positional and geometric isomers of linoleic acid with two conjugated double bonds, of which the cis9,trans11 isomer is the most abundant natural isomer with biological activity, accounting for 75–90% of total CLA in ruminant meat [52]. CLA is mainly deposited in triacylglycerols, and its levels in beef lipids is influenced by intrinsic factors such as breed, sex, age, fat content, and type of muscle [53]. In addition, it is influenced by feeding conditions, with grazing and supplementation with oilseeds, vegetable oils, and fish oil having been shown to be enhancing factors [54].

However, in the present study, supplementing the diet of young Simmental bulls with whole linseed had no effect (*p* > 0.05) on CLA content (Table 3). This is consistent with previous reports in which supplementation with whole linseed at various levels showed no effect on the intramuscular CLA content, measured either in total lipids or in lipid fractions (polar or neutral), of grain-fed young bulls, including the Simmental breed [9,55]. In contrast, several studies reported a significant increase in CLA content in purebred [36] or crossbred bulls [24] when fed extruded linseed, suggesting better rumen digestion of the extruded seeds. As argued by Doreau and Ferlay [22], this is possibly due to the higher oil release rate of extruded seeds into the rumen compared to whole seeds, which could lead to increased production of trans-FA in the rumen. On the other hand, seed coat might protect PUFA from rumen biohydrogenation and increase the passage of PUFA to the duodenum [5]. However, the effects of feeding with linseed on the CLA content of beef remain unclear and require further investigation.

There was a significant effect of diet on C20:0 (*p* < 0.01) and total SFA (*p* < 0.05) contents (Table 3), which were reduced on average by 10% and 3% by linseed supplementation compared to the control diet. For C14:0 and C15:0, a significant (*p* < 0.05) interaction between diet and age at slaughter existed, with lower values (*p* < 0.05) for C14:0 and a similar trend (*p* = 0.076) for C15:0 detected only in animals fed linseed and slaughtered at a younger age. Compared to other studies in which grain-fed young bulls were supplemented with linseed, the observed decrease in SFA in the total lipids of beef agrees with the results of Morittu et al. [24], while in the studies of Albertí et al. [15] and Conte et al. [36] SFA content was not significantly affected by linseed supplementation. In the study by Corrazzin et al. [9], in which the polar and neutral lipid fractions were analysed separately, the effect of the linseed diet on SFA was also not confirmed, although the difference in SFA content in polar lipids (i.e., triglycerides) between the linseed and control diets was evident (53.57 vs. 58.07 g/100 g total lipids, *p* = 0.052). Feeding with high-corn diets may decrease the protozoal and bacterial populations responsible for rumen biohydrogenation by reducing the rumen pH [56]. Hence, the significant effect on the beef SFA observed in the present study may be at least partly related to the higher content of corn in the bulls’ diet than in other studies, which may favour a reduction in beef SFA.

The contents of MUFA, n-6 FA, and total PUFA in the LT muscle were not significantly (*p* > 0.05) affected by linseed supplementation (Table 3). As expected, the effect of diet was significant for n-3 PUFA. EPA was increased (*p* < 0.05) by the linseed supplimentation compared to the control diet in both age groups, while for ALA (C18:3n-3) and C22:5n-3, as well as for total n-3 PUFA, a strong effect of diet (*p* < 0.001) was associated with a significant interaction (*p* < 0.05) with slaughter age. Compared to the control diet, the concentration of these n-3 FA was increased (*p* > 0.05) by linseed supplementation in the bulls slaughtered at a younger age (i.e., 0.28 vs. 0.56 for ALA, 0.25 vs. 0.28 for C22:5 n-3, and 0.67 vs. 1.27 g/100 g total FA for total n-3). However, when the bulls were slaughtered at an older age, the effects of a linseed diet on these FAs were less pronounced and were only observed for ALA, but to a lesser extent than in the younger counterparts (i.e., 0.31 vs. 0.44 g/100 g total FA).

Linseed is an oilseed rich in ALA (e.g., 51.9%, Supplementary Materials Table S1), and in all trials where linseed (whole or extruded) was supplemented in the diet of grain-fed young bulls, a significant increase in ALA and total n-3 PUFA in beef FA was demonstrated [9,15,24,36,55]. However, the ability to modify the FA profiles of beef through diet is substantially limited, as the rumen microbiota biohydrogenates a considerable amount of unsaturated FA from the diet. The biohydrogenation of ALA and other dietary PUFAs (e.g., C18:2n-6) involves the rapid and extensive hydrolysis of esterified dietary fats and oils and the subsequent conversion of released unsaturated FA into SFA, particularly in 18:0, by isomerisation to trans-FA intermediates and subsequent hydrogenation of the double bonds [57]. The biohydrogenation of unsaturated FA in the rumen also results in a wide range of isomers of PUFA and MUFA, in particular trans and conjugated FA [58]. The quantity and composition of these biohydrogen products in beef depends largely on the type and the amount of FA supplied in the diet, with C18:2n-6 (e.g., from grains) generally being more incompletely biohydrogenated than ALA; and rumen conditions (e.g., pH), which influence the degree of biohydrogenation [56,59].

Hence, despite the positive responses to linseed supplementation observed in present and other studies with grain-fed young bulls, the contribution of n-3 PUFA to the FA profile of beef remains generally low. For example, in the intramuscular fat of young bulls fed a linseed diet, the proportion of ALA and total n-3 PUFA ranged from 1.34% to 1.62% and 2.45% to 2.48% for whole-linseed supplementation [9,15] and from 1.26% to 1.7% and 2.49% to 4.05% for extruded-linseed supplementation [24,36]. The proportion of longer-chain n-3 derivatives of ALA detected in these experiments was much lower regardless of diet (e.g., for EPA, between 0.2 and 0.4), probably due to the complexity of the enzyme system involved in the ALA-to-C20-22 n-3 conversion process, which consists of desaturases and elongases [60]. EPAs (and DHAs when detected), which are considered particularly beneficial for human health, were only moderately increased by linseed supplementation [9,24] or remained unchanged [15,36].

Compared to other studies, the lower proportions of ALA, EPA, and total n-3 PUFA in intramuscular fat observed in the present work are probably related to the lower levels of linseed supplementation than in other studies, which were apparently not sufficient for a more significant increase in n-3 PUFA, especially in more-grown-up and heavier animals. It is worth noting that the bulls slaughtered at 17 months of age in this experiment were the heaviest (629.4 kg average live weight) of all bulls analysed in the aforementioned studies, which may also have contributed to a lower response to linseed feed in the present study.

### 3.5. Nutritional Indices

Dietary fats are known to affect plasma lipid levels and lipoprotein and cholesterol metabolism, which have a significant impact on the maintenance of normal vascular wall function and the development of pathological changes, atherosclerosis, and thrombosis [61]. Dietary SFA may contribute to the development of cardiovascular disease by increasing total blood cholesterol levels. Conversely, an increased intake of PUFAs, particularly LA and ALA, lowers total cholesterol levels, while MUFAs such as C18:1 have little or no effect in this regard. Therefore, the PUFA-to-SFA ratio (P/S) is often used as an indicator of the quality of dietary fats in relation to atherogenicity in humans. The nutritionally recommended value for this ratio is ≥0.4 [62]. The P/S ratio in beef is generally lower than recommended due to the biohydrogenation of unsaturated FA in the rumen [63], and this was also demonstrated in the present study, regardless of diet or age at slaughter. In addition, increase in slaughter weight or linseed supplementation had no significant effect (*p* > 0.05) on P/S ratio. However, in bulls slaughtered at a younger age, the P/S ratio when fed linseed was closer to the recommended threshold and was more favourable than the P/S ratio in other groups, which is consistent with the magnitude of the dietary effect observed for SFA content.

Another measure of the nutritional quality of dietary fats is the index of atherogenicity (AI), proposed by Ulbricht and Southgate [50]. The AI is considered particularly useful as it includes neutral MUFA and puts emphasis on C14:0, which is considered to have the most harmful cardiovascular effect [62]. In the present study, a decrease in AI (on average by 7.5%) was achieved by a change in diet (*p* < 0.05), while slaughter age had no effect (*p* > 0.05). More favourable results (i.e., lower AI) were again observed in younger bulls, the only group in which a significant decrease in C14:0 was observed when supplied with linseed (SA × Dinteraction, *p* < 0.05).

PUFAs of different n-series have different structures and metabolic effects, with n-3 PUFAs playing a more favourable role in the modulation of inflammatory reaction, vasodilatation, and thrombosis tendency than n-6 PUFAs [64]. To prevent diet-related chronic diseases, it was therefore recommended that the intake of n-3 PUFAs be increased in relation to n-6 PUFAs, whereby the optimal ratio of n-6 to n-3 in human diet should be less than 4 [62].

In the present study (Table 3), both age at slaughter and diet had a significant effect (*p* < 0.001) on the n-6/n-3 ratio in the intramuscular fat of young bulls, with indices being slightly higher in older animals (by 2.4% on average) and considerably lower in linseed-fed animals (by 37.7% on average). However, the n-6/n-3 ratios reported in the present study for bulls fed linseed (around 10 to 13 at lower or higher slaughter ages, respectively) are well above the dietary recommendations and were generally higher than those found in similar studies [15,24,36,55].

### 3.6. Oxidative Stability

Table 4 shows the effects of slaughter age (13 vs. 17 months) and diet (control vs. linseed-enriched) on TBARS values, a measure of lipid oxidation, in LT muscle during storage (at days 0, 3, and 6).

Lipid oxidation is a key factor contributing to the spoilage of refrigerated stored meat, significantly affecting sensory quality and influencing consumer perception of beef [65]. The results of our study (Table 4) show that lipid oxidation increased progressively over time in all groups, which is consistent with the natural oxidation process during meat storage [19,66]. Despite the increase, TBARS values remained below the threshold of 2 mg MDA/kg of meat, which is widely regarded as the level at which rancidity becomes perceptible to consumers [67]. Studies indicate that most consumers can detect rancidity in meat when TBARS values reach or exceed 2 mg MDA/kg [67,68]. Campo et al. [65] also pointed out that the sensory profile of beef changes significantly at TBARS levels above 2.28 mg MDA/kg, with rancid off-flavors overpowering the natural beef flavor.

Meat from older bulls (17 months of age) had significantly lower TBARS values across all storage days than that of younger bulls (13 months of age), indicating improved oxidative stability. While there is little literature directly linking age to oxidative stability in beef, the results of this study suggest that the lower lipid oxidation observed in older bulls may be due to their higher IMF content. In muscle lipids, the proportion of SFA-rich triglycerides increases relative to PUFA-rich phospholipids with an increase in IMF content [69]. This higher content of saturated fat likely dilutes the concentration of PUFAs, which are more susceptible to oxidation, and thus increases oxidative stability. In addition, muscle maturity in older bulls improves the structural integrity of muscle fibers, which could reduce the activity of pro-oxidant enzymes [70]. The presence of natural antioxidants [71] and differences in muscle fiber composition [72] also contribute to better oxidative stability. These combined factors could contribute to the reduced lipid oxidation and lower TBARS values observed in older animals.

On the contrary, the results show that the linseed-enriched diet led to higher TBARS values (*p* < 0.05). While neither linoleic acid (C18:3n-6) nor total PUFA content in the LT muscle showed statistically significant differences (*p* < 0.05) between the dietary groups (see Table 3), the linseed diet shows a noticeable efficiency for higher ALA and n-3 PUFA levels. This increase, though not statistically significant for total n-3 PUFA content in bulls slaughtered at 17 months of age, suggests that linseed supplementation effectively enriched the muscle with these beneficial fatty acids, but not to a level sufficient to reach significance in more-grown-up animals. The observed lack of significant differences in PUFA content between the dietary groups suggests that factors other than absolute PUFA levels may have contributed to higher lipid oxidation. Although the total PUFA content in the LT muscle was not statistically influenced by diet, the susceptibility of PUFAs to oxidation still plays a crucial role in oxidative stability. This indicates that even small amounts of highly oxidizable fatty acids like ALA can influence TBARS values when combined with pro-oxidative conditions, such as the absence of effective antioxidant defenses such as vitamin E, which was not administered in the present study. Corazzin et al. [9] and Hou et al. [17] support the idea that PUFA-rich diets, including those supplemented with linseed, can enhance lipid oxidation even without significant increases in muscle PUFA concentration.

Moreover, the TBARS results suggest that while linseed supplementation enhances the nutritional quality of beef by enriching it with n-3 FA, it can also increase oxidative stress. As noted by Bartkovsky et al. [16], the natural antioxidants present in linseed, such as lignans, may not fully counteract this effect, necessitating additional antioxidative strategies, such as incorporating exogenous antioxidants or improving handling and storage practices. For example, in the study by Morittu et al. [24], linseed supplementation was combined with vitamin E enrichment to evaluate its impact on oxidative stability in beef. They found that the addition of vitamin E significantly reduced TBARS values, improving both the oxidative stability and the shelf life of PUFA-enriched meat. This emphasizes the importance of such interventions in maintaining meat quality during storage while meeting consumer demand for healthier, longer-lasting products.

The interaction between slaughter age and diet had no statistically significant effect (*p* > 0.05) on the oxidative stability of LT muscle.

## 4. Conclusions

The present study has shown that low-level linseed supplementation (around 1%) throughout the fattening period in grain-fed Simmental bulls slaughtered at either 13 or 17 months of age can increase the dressing percentage at both ages of slaughter, without negative effects on meat quality. The intramuscular fat of linseed-fed bulls was generally less saturated, while the proportion of beneficial n-3 PUFAs increased only up to 13 months of age. Nutritional indices such as the atherogenic index and n-6/n-3 ratio were reduced by linseed supplementation but remained generally unfavorable for human diets. Lipid oxidation, measured by the TBARS test, was generally more pronounced in younger and linseed-fed animals. Overall, these results suggest that linseed supplementation can improve the FA composition of beef, but that higher levels, especially in older animals with advanced rumen digestion, and antioxidant strategies to counteract increased lipid oxidation may be required to optimize meat stability and nutritional value. Sensory evaluation of the meat should be included in future studies.

## Figures and Tables

**Table 1:** Ingredients and chemical composition of diets.

	Up to 13 Months		After 13 Months	
	Control Diet	Linseed Diet	Control Diet	Linseed Diet
Ingredients (kg per head daily):				
High-moisture maize	6.4	6.2	7.7	7.5
Maize silage	8.1	7.9	9.0	8.8
Protein-rich feed (34%) ^a^	1.4	1.4	1.5	1.4
Hay	0.23	0.23	0.23	0.23
Whole linseed grain	-	0.13	-	0.16
Chemical composition (g/kg DM):				
Dry matter (DM)	589	565	609	605
Crude protein	129	127	127	126
Ether extract	38	43	35	39
Neutral detergent fibre	162	170	169	179
Acid detergent fibre	72	77	75	81
Ash	46	45	39	43
pH value	4.45	4.35	4.28	4.26
Fatty acid composition (%) ^b^:				
C12:0	0.10	0.09	0.08	0.08
C12:1	0.34	0.38	0.27	0.30
C14:0	0.17	0.15	0.16	0.16
C16:0	12.76	11.40	12.95	11.32
C16:1	0.30	0.22	0.32	0.24
C18:0	3.48	3.35	2.59	2.65
C18:1	26.57	25.01	26.91	24.40
C18:2n-6	50.49	45.63	51.92	44.42
C18:3n-3	3.22	11.52	2.57	14.37
C20:0	0.46	0.40	0.36	0.31
Total SFA	18.20	16.55	17.42	15.37
Total MUFA	27.96	26.19	27.97	25.37
Total PUFA	53.84	57.26	54.61	59.27

^a^ Based on soybean meal with a mineral and vitamin additive, which comprised (on a DM basis): 34% crude protein, 2% crude fat, 8.5% crude fiber, 16.5% ash, 30,000 U of vitamin A, 3300 U of vitamin D3, 120 mg of vitamin E/kg, 37.5 mg Cu/kg. ^b^ Percentage of total fatty acids quantified.

**Table 2:** Effect of slaughter age (13 vs. 17 months) and diet (control vs. linseed) on the slaughter performance and meat quality traits of Simmental bulls.

Traits	13 Months		17 Months		RMSE	Significance ^a^		
	Control (C13)	Linseed (L13)	Control C17)	Linseed (L17)		SA	D	SA × D
n	20	20	20	20				
Final weight (kg)	552.5	556.5	632.1	626.7	40.7	***	ns	ns
Average daily gain (kg)	1.5	1.5	1.2	1.2	0.2	***	ns	ns
Hot carcass weight (kg)	317.3	327.7	374.8	376.1	25.6	***	ns	ns
Dressing percentage (%)	57.4	58.9	59.3	60.0	1.4	***	**	ns
Trimmed fat ^b^ (kg)	5.3	5.1	7.2	7.5	2.8	***	ns	ns
EUROP score ^c^	3.9 ^b^	4.4 ^a^	4.7 ^a^	4.7 ^a^	0.6	***	*	*
Fatness score ^d^	3.0	2.9	3.1	3.1	0.3	*	ns	ns
*M. longissimus thoracis*								
pH_1_	6.49	6.32	6.62	6.43	0.38	ns	*	ns
pH_2_	5.73	5.70	5.68	5.77	0.34	ns	ns	ns
CIE L* (lightness)	41.20	41.45	39.22	38.67	2.43	***	ns	ns
CIE a* (redness)	23.48	23.50	23.65	23.23	1.31	ns	ns	ns
CIE b* (yellowness)	8.64	8.88	8.44	7.78	1.16	*	ns	ns
Moisture (g/kg)	747.8	746.9	743.8	742.7	11.34	ns	ns	ns
Protein (g/kg)	221.8	225.6	222.6	223.3	5.05	ns	*	ns
Intramuscular fat (g/kg)	23.4	20.4	28.0	28.3	9.47	**	ns	ns
Ash (g/kg)	10.5	10.6	10.4	10.5	0.20	*	*	ns

n = number of animals. ^a^ Significance of main effects (SA—slaughter age, D—diet) and their interaction (SA × D): * *p* < 0.05; ** *p* < 0.01; *** *p* < 0.001; ns—not significant; for significant SA × Dinteraction, the least squares means with different letters within a row differ significantly according to the Tukey post hoc test (*p* ≤ 0.05). RMSE—root mean square error. ^b^ Carcass separable fat. ^c^ 1 = poor (P) to 5 = excellent (E). ^d^ 1 = minimum to 5 = maximum.

**Table 3:** Effect of slaughter age (13 vs. 17 months) and diet (control vs. linseed) on fatty acid profile (g/100 g of total fatty acids) of *M. longissimus thoracis* of Simmental bulls.

	13 Months		17 Months		RMSE	Significance ^a^		
	Control C13)	Linseed (L13)	Control (C17)	Linseed (L17)		SA	D	SA × D
C12:1	0.67	0.55	0.59	0.57	0.27	ns	ns	ns
C14:0	2.94 ^a^	2.52 ^b^	2.89 ^ab^	2.93 ^a^	0.50	ns	ns	*
C14:1	0.45	0.39	0.41	0.41	0.17	ns	ns	ns
C15:0	0.43	0.36	0.36	0.39	0.08	ns	ns	*
C16:0	23.72	22.63	24.22	23.78	2.00	ns	ns	ns
C16:1	2.85	3.01	2.84	2.82	0.54	ns	ns	ns
C17:0	1.11	0.99	0.95	0.99	0.20	ns	ns	ns
C17:1	1.00	0.92	0.85	0.88	0.18	*	ns	ns
C18:0	17.46	16.64	16.87	16.72	1.60	ns	ns	ns
C18:1	37.55	37.28	37.76	38.46	3.36	ns	ns	ns
C18:2n-6 LA	7.05	8.63	8.07	7.76	2.99	ns	ns	ns
C18:3n-3 ALA	0.28 ^b^	0.56 ^a^	0.31 ^bc^	0.44 ^a^	0.14	ns	***	*
C18:2 (c + t) CLA	0.21	0.22	0.29	0.33	0.07	***	ns	ns
C20:0	0.11	0.09	0.10	0.10	0.01	ns	**	ns
C20:1	0.26	0.25	0.27	0.27	0.04	ns	ns	ns
C20:2n-6	0.17	0.13	0.17	0.13	0.08	ns	ns	ns
C20:3n-6	0.44	0.51	0.44	0.39	0.20	ns	ns	ns
C20:4n-6 AA	2.18	2.70	2.11	1.93	1.01	ns	ns	ns
C20:5n-3 EPA	0.10	0.17	0.06	0.09	0.08	**	*	ns
C22:4n-6	0.40	0.45	0.38	0.31	0.14	**	ns	ns
C22:5n-3	0.25 ^b^	0.48 ^a^	0.21 ^b^	0.29 ^b^	0.15	***	***	*
ΣSFA	45.87	43.42	45.44	44.95	2.33	ns	**	ns
ΣMUFA	42.89	42.55	42.51	43.41	3.62	ns	ns	ns
ΣPUFA	11.24	14.03	12.05	11.64	4.61	ns	ns	ns
Σn-6	10.36	12.54	11.20	10.51	4.35	ns	ns	ns
Σn-3	0.67 ^b^	1.27 ^a^	0.56 ^b^	0.80 ^b^	0.33	***	***	*
n-6/n-3	15.64	9.80	20.96	13.02	3.01	***	***	ns
P/S	0.25	0.33	0.27	0.26	0.12	ns	ns	ns
AI	0.66	0.57	0.66	0.65	0.09	ns	*	ns

^a^ Significance of main effects (SA—slaughter age, D—diet) and their interaction (SA × D): * *p* < 0.05; ** *p* < 0.01; *** *p* < 0.001; ns—not significant; for significant SA×Dinteraction, the least square means with different letters within a row differ significantly according to Tukey’s post hoc test (*p* ≤ 0.05). RMSE—root mean square error. ΣSFA—saturated fatty acids = (C11:0 + C12:0 + C13:0 + C14:0 + C15:0 + C16:0 + C17:0 + C18:0 + C19:0 + C20:0 + C22:0 + C24:0). ΣMUFA—monounsaturated fatty acids = (C12:1 + C13:1 + C14:1 + C15:1 + C16:1 + C17:1 + C18:1 + C19:1 + C20:1 + C22:1 + C24:1). ΣPUFA—polyunsaturated fatty acids = (C18:2n-6 + C18:3n-6 + C18:2n-7 + C20:2n-6 + C20:3n-6 + C20:4n-6 + C22:2n-6 + C22:4n-6 + C22:5n-6 + C18:3n-3 + C20:3n-3 + C20:5n-3 + C22:5n-3 + C22:6n-3). P/S = PUFA/SFA. AI = atherogenic index = [C12:0 + (4*C14:0) + C16:0]/[(∑ MUFA) + (∑ PUFA)], Ulbricht and Southgate [50].

**Table 4:** Effect of slaughter age (13 vs. 17 months) and diet (control vs. linseed) on TBARS values (mg MDA/kg tissue) of *M. longissimus thoracis* of Simmental bulls.

TBARS	13 Months		17 Months		RMSE	Significance ^a^		
	Control (C13)	Linseed (L13)	Control (C17)	Linseed (L17)		SA	D	SA × D
Day 0	0.24	0.35	0.06	0.12	0.10	***	**	ns
Day 3	0.74	1.18	0.13	0.36	0.55	***	*	ns
Day 6	1.04	1.68	0.22	0.52	0.73	***	*	ns

^a^ Significance of main effects (SA—slaughter age, D—diet) and their interaction (SA × D): * *p* < 0.05; ** *p* < 0.01; *** *p* < 0.001; ns—not significant. TBARS = 2-thiobarbituric acid-reactive substances, MDA = malonaldehide. RMSE—root mean square error.

## Data Availability

The data that support the findings of this study are available from the corresponding author, D.K., upon reasonable request due to the legal reasons.

## Supplementary Materials

The following supporting information can be downloaded at: https://www.mdpi.com/article/10.3390/foods14071098/s1, Table S1: Average chemical and FA composition of diet components provided to bulls.
