# Supplementary material for: Effect of Linseed Feeding on Carcass and Meat Quality and Intramuscular Fatty Acid Profile of Simmental Bulls Slaughtered at Different Ages
Source: Foods. 2025 Mar 21;14(7):1098. doi: 10.3390/foods14071098 (PMC11988707; doi:10.3390/foods14071098)
Supplement: Supplementary file 1 [file foods-14-01098-s001.zip › foods-3496598-supplementary.pdf]

Article

Effect of Linseed Feeding on Carcass and Meat Quality and Intramuscular Fatty Acid Profile of Simmental Bulls Slaughtered at Different Ages

Ana Kaić, Dubravko Škorput, Zoran Luković, Krešimir Salajpal, Kristina Kljak, Nives Marušić Radovčić and Danijel Karolyi

Supplementary Data, Table S1

Table S1. Average chemical and FA composition of diet components provided to bulls

	HMC	CS	PS[a]	H	WL
Chemical composition (g/kg DM):					
Dry matter (DM)	745	416	925	935	929
Crude protein	79	69	366	67	192
Ether extract	45	32	19	19	237
Neutral detergent fibre	124	400	148	632	235
Acid detergent fibre	31	204	88	356	139
Ash	15	45	181	60	48
Fatty acid composition (%)[b]:					
C12:0	0.02	0.27	0.04	0.76	0.01
C12:1	0.25	0.41	0.40	1.36	0.04
C14:0	0.07	0.34	0.22	1.28	0.05
C16:0	12.41	13.74	11.58	27.02	5.38
C16:1	0.21	0.59	1.02	0.47	0.07
C18:0	2.74	3.52	4.30	8.13	3.01
C18:1	28.22	25.76	36.73	4.82	20.83
C18:2n-6	52.60	47.09	37.55	17.74	17.26
C18:3n-3	1.56	5.04	5.62	26.70	51.88
C20:0	0.44	0.61	0.33	1.55	0.10
Total SFA	16.50	20.20	17.55	46.24	9.39
Total MUFA	29.25	27.30	39.14	7.62	21.22
Total PUFA	54.25	52.50	43.31	46.13	69.39

HMC: high moisture corn, CS: corn silage, PS: protein supplement, H: hay, WL: whole linseed

[a] Supplied (on DM basis): 30 000 U of vitamin A, 3 300 U of vitamin D3, 120 mg of vitamin E/kg, 37.5 mg Cu/kg.

[b] Percentage of total fatty acids quantified.
